# Systematic review of worldwide trends in assisted reproductive technology 2004–2013

**DOI:** 10.1186/s12958-016-0225-2

**Published:** 2017-01-10

**Authors:** Vitaly A. Kushnir, David H. Barad, David F. Albertini, Sarah K. Darmon, Norbert Gleicher

**Affiliations:** 1 The Center for Human Reproduction, 21 East 69th Street, New York, NY 10021 USA; 2 Wake Forest School of Medicine, Winston-Salem, NC USA; 3 Foundation for Reproductive Medicine, New York, NY USA; 4 University of Kansas Medical Center, Kansas City, KS USA; 5 University of Vienna School of Medicine, Vienna, Austria; 6 The Rockefeller University, New York, NY USA

**Keywords:** In vitro fertilization, Assisted reproductive technology, Single embryo transfer, Live birth rates, Embryo cryopreservation

## Abstract

**Background:**

Assisted Reproductive Technology (ART) has undergone considerable changes over the last decade, with consequences on ART outcomes in different regions of the world being unknown.

**Methods:**

We conducted a systematic review of published national and regional ART registry data to assess how changes in clinical practice between 2004 and 2013 have impacted outcomes in Australia and New Zealand, Canada, Continental Europe, the United Kingdom (U.K.), Japan, Latin America, and the United States (U.S.). The data reflect 7,079,145 total ART cycles utilizing both fresh and previously cryopreserved embryos from autologous oocytes that resulted in 1,454,724 live births. This review focused on the following measures: ART cycle volume, use of cryopreserved embryos, single embryo transfer (SET), live birth rates in fresh and frozen-thawed cycles, and perinatal outcomes in recent years.

**Results:**

SETs and utilization of frozen-thawed embryos increased worldwide over the study period. In 2012 SET utilization in all ART cycles was highest in Japan and Australia/New Zealand (82.6% and 76.3% respectively) and lowest in Latin America (16.0%). While gradual improvements in live birth rates were observed in most regions, some demonstrated declines. By 2012–2013, fresh cycle live birth rates were highest in the U.S. (29%) and lowest in Japan (5%). In Japan, the observed decline in fresh cycle live birth rate coincided with transition to minimal stimulation protocols, transfer of frozen-thawed rather than fresh embryos, and implementation of an SET policy. Similarly, implementation of an SET policy in parts of Canada was followed by a decline in fresh cycle live birth rate. Increasing live birth rates in frozen-thawed embryo cycles, seen all over the world, partially compensated for declines in fresh ART cycles. During 2012–2013 Australia/New Zealand and Japan reported the lowest multiple delivery rates of 5.6 and 4% respectively while the US had the highest of 27%. In recent years, preterm delivery rates in all regions ranged between 9.0 to 16.6% for singletons, 53.9 to 67.3% for twins, and 91.4 to 100% for triplets and higher order multiples. Inconsistencies in the way perinatal outcome data are presented by various registries, made comparison between regions difficult.

**Conclusions:**

ART practices are characterized by outcome differences between regions. International consensus on the definition of ART success, which accounts for perinatal outcomes, may help to standardize worldwide ART practice and improve outcomes.

**Trial registration:**

PROSPERO (CRD42016033011)

**Electronic supplementary material:**

The online version of this article (doi:10.1186/s12958-016-0225-2) contains supplementary material, which is available to authorized users.

## Background

The last decade has witnessed a number of new treatments integrated into routine assisted reproductive technology (ART) practice, at times making public outcome reporting more challenging and less transparent [1, 2]. A number of new IVF practice regimens have been applied differently over the world and beg for further critical evaluation including use of routine blastocyst-stage in place of cleavage-stage embryo transfer [3] replacement of fresh embryo transfer by embryo cryopreservation (“freezing”) and subsequent thawed embryo transfer [4], preimplantation genetic screening (PGS), [5] single embryo transfer (SET) in place of double embryo transfer (DET) [6] and minimal stimulation protocols [7, 8]. At times spurred by government policies or local recommendations of professional societies, adoption of some practice changes occurred faster in some parts of the world than others. The most recent worldwide report on ART practices was authored by the International Committee for Monitoring Assisted Reproductive Technology (ICMART), and was based on data from 2008 through 2010 [9].

Here we examined longitudinal data, reported by ART centers between 2004 and 2013 to regional registries worldwide. Our objectives were to study longitudinal changes in ART practice over the last decade of reported data, including cycle volumes, use of previously cryopreserved embryos and of SET. In addition, we assessed ART outcomes, based on live birth rates in fresh and frozen-thawed cycles. By comparing practice patterns and outcomes across regions, as well as longitudinal changes within regions, this review offers insights into the global evolution of ART practice.

## Methods

This systematic review was exempted from IRB approval because only publically available de-identified data were used for the analyses.

### Search strategy and data collection

This systematic review was registered with PROSPERO (CRD42016033011) and is reported in accordance to PRISMA (Additional file 1: Figure S1, checklist). We systematically examined aggregate data publically available from national and regional ART registries since 2004. ART registries were identified primarily via member and associated fertility societies of the International Federation of Fertility Societies [10].

In order to standardize aggregate data from different regions, we only included reports which allowed calculation of number of live births per initiated fresh autologous ART cycles (i.e. utilized the patient’s own oocytes) over multiple years between 2004 and 2013. In all we were able to identify ten ART registries with publically available data. Annual regional ART registry reports which permitted calculation of live birth per initiated cycle were identified for seven registries: Australia and New Zealand combined, [11] Canada, [12] Continental Europe, [13] the U.K., [14] Japan [15] Latin America [16] and the U.S. [17] (Table 1). Three other registries (South Korea, [18] Israel, [19] and South Africa [20]) did not permit this calculation, and were therefore excluded from assessment. Live birth rates in frozen-thawed ART cycles are calculated per initiated cycle for all regions except Latin America which reports data per embryo transfer and Europe which reports data per thaw procedure. Frozen-thawed ART cycles were undertaken for various indications including cryopreservation of surplus embryos following a fresh transfer, as well as, embryo banking for PGS, due to ovarian hyperstimulation and fertility preservation. These cycles were analyzed together since specific indications could not be determined from the registry data.

**Table 1:** Total number of ART cycles and live births reported for each region

	Fresh Embryo Cycles	Frozen Embryo Cycles	Live Births
Australia and New Zealand^a^	357,494	207,678	101,012
Canada^a^	111,417	44,707	37,373
Europe^c^	1,959,613	605,673	548,889
Japan^b^	1,199,715	585,670	224,170
Latin America^a^	265,316	51,047	65,849
UK^d^	335,438	78,730	101,364
US^a^	995,410	281,237	376,067
Total	5,224,403	1,854,742	1,454,724
	7,079,145		

Data for years: ^a^2004–2013, ^b^2004–2012, ^c^2004–2011, ^d^2006–2013

### Data quality and validation

The data in Australia and New Zealand Assisted Reproduction Database (ANZARD) are self-reported by fertility centers, and validated by the National Perinatal Epidemiology and Statistics Unit (NPESU), with data queries being followed up with fertility center staff. [11] Reporting of ART cycles in Australia is a requirement for fertility centers to be licensed by the Reproductive Technology Accreditation Committee. The Fertility Society of Australia helps ensure the quality of data by validating selected records against clinic files during annual inspections. In 2012, ART data were collected from 37 fertility centers. Outcomes were not provided for only 1.6% of clinical pregnancies which were lost to follow up.

Participation of fertility centers in the Canadian Assisted Reproductive Technologies Register (CARTR) is voluntary [12]. In 2012, 32 out of 33 centers in Canada submitted data to CARTR where they were manually checked for accuracy, and clarifications were requested from centers. No on-site data validation from source documents was performed.

Data for 2011 from Europe comes from 33 out of 51 countries, and a total of 1064 fertility centers. Among these 33 countries, 17 reported complete data sets, and 16 only partial ones [13]. Reporting was compulsory for 17 countries and voluntary for 16. Data were collected in 14 countries by a National Health Authority, in 17 by a Medical Organization, and in 2 by private initiative. Only 13 countries reported some kind of data validation process.

European reports include data from the U.K., which we here analyzed and reported separately. The 2013 data from the U.K. was reported under legal mandate by 78 fertility centers to the Human Fertilization and Embryology Authority (HFEA), which validated the data [14].

The Japan Society of Obstetrics and Gynecology (JSOG) has required all centers that perform ART to register cycle-specific information since 2007 [15]. In 2012, 586 of 589 (>99%) registered ART centers reported data.

The 2013 report from Latin American Registry of Assisted Reproduction (RLA) is based on voluntarily reported data from 158 institutions in 15 countries, covering more than 80% of ART cycles performed in the region [16]. Patient data from this region have been collected and validated since 2010. Earlier RLA reports were based on summary data obtained from participating fertility centers in 12 countries.

The 2012, U.S. source data were self-reported by 456 fertility centers under legal mandate to the Centers for Disease Control and Prevention (CDC), and covered more than 97% of all ART cycles performed in the country [17]. Select on-site annual validation visits including chart reviews have found low discrepancy rates (<5%) for most variables in recent years.

### Outcome measures

This study includes analysis of longitudinal trends in volume of fresh and frozen-thawed autologous ART cycles, as well as, utilization of SET for each region. The primary outcomes of interest were live birth rates in fresh and frozen-thawed cycles, calculated for each region for each year of available data. Additionally, perinatal outcomes were reviewed for the recent years of registry data. Live births included single and multi-fetal deliveries. Our interpretation of findings refers to absolute differences in data within and between regions; statistical testing of comparisons between regions was not performed.

## Results

### ART cycle volume

**Fig. 1:**
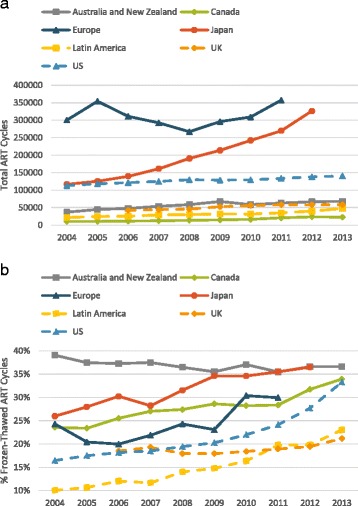
**a** Total volume of autologous oocyte ART cycles for study period 2004–2013. The figure demonstrates gradually increasing ART cycle number in most regions, except for Japan, which demonstrates a rapid increase in cycle number. European cycle volume fluctuated depending on number of reporting countries in that region each year. **b** Proportion of ART cycles which utilized frozen-thawed embryos created from autologous oocytes. The figure demonstrates that utilization of previously cryopreserved embryos has increased in all regions except Australia/New Zealand

Figure 1a demonstrates that the volume of ART cycles in most studied regions moderately increased. The only exception was Japan where cycle volume nearly tripled during the study period. As Fig. 1b demonstrates, utilization of previously cryopreserved embryos increased in all regions except Australia/New Zealand, where already a decade ago such cycles were more common than in any other region, accounting for more than 35% of all ART.

### Single embryo transfer (SET)

**Fig. 2:**
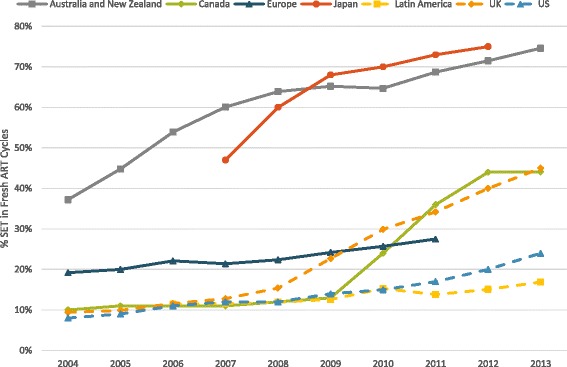
Proportion of autologous fresh embryo cycles in which SET was used. The figure demonstrates increasing utilization of SET in all studied regions

In fresh ART cycles, SET utilization increased in all regions (Fig. 2), now representing more than half of all ART cycles in Japan and in Australia/New Zealand. Utilization of SET in frozen-thawed ART cycles, likewise, increased over the study period in the four regions which reported this data. In Australia and New Zealand SET in frozen-thawed ART cycles increased from 45.3% in 2004 to 84.9% in 2013; in Japan from 55% in 2007 to 80% in 2012; in Latin America from 10% in 2008 to 19.9% in 2013; and in Canada from 18% 2004 to 55.2% in 2013. In Canada SET utilization in both fresh and frozen-thawed ART cycles increased rapidly following implementation of a SET policy in Quebec in 2009.

### ART live birth rates

**Fig. 3:**
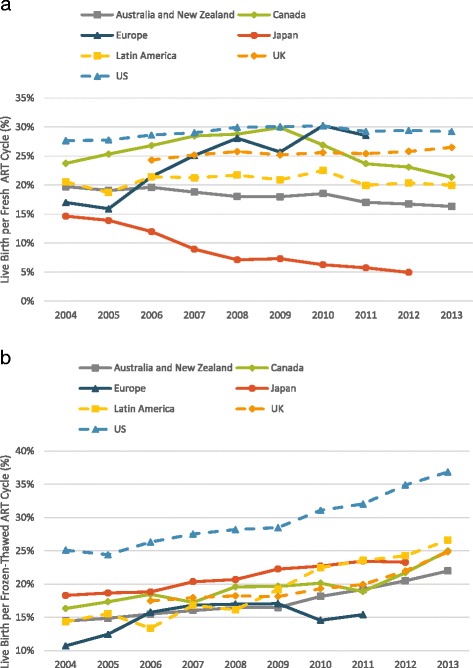
**a** ART live birth rates with fresh embryos created from autologous oocytes. The figure demonstrates stable or slightly decreasing live birth rates in most regions. Increasing live birth rates are noted in continental Europe while most pronounced decreases are noted in Japan and in Canada after 2009. **b** ART live birth rates with frozen-thawed embryos created from autologous oocytes. The figure demonstrates improving live birth rates in frozen-thawed cycles in all regions. Data is reported per initiated cycles for all regions except Latin America which reports data per embryo transfer and Europe which reports data per thawing procedure

Live birth rates in fresh ART cycles varied considerably between regions. By 2012–2013, the last years for which national data have been reported, they were highest in the U.S. (29%) and lowest in Japan (5%) (Fig. 3a).

In fresh ART cycles, live birth rates in most parts of the world remained flat or minimally increased. Likely the most remarkable finding of this part of the study was the precipitous decline in fresh cycle live birth rates in Japan between 2004 and 2012 (Fig. 3a). During this period utilization of SET increased in Japan as demonstrated in Fig. 2; additionally, the practice in that country transitioned away from fresh embryo transfer and toward transfer of frozen-thawed embryos as demonstrated in Fig. 1b [7, 8, 21]. By 2012, only 27% of all ART live birth in Japan were from fresh cycles and 73% were from frozen-thawed cycles.

That increasing utilization of SET may be associated with declining live birth rates is also suggested by the combined data set from Australia/New Zealand, where live delivery rates per initiated fresh cycle gradually declined to 16.3% in 2013 [11]. Similarly, starting in 2009, a government mandated SET practice was implemented in the populous Canadian province of Quebec (Fig. 2); this was followed by a progressive decline in fresh cycle live birth rate during subsequent years (Fig. 3a) [22, 23].

Another notable finding was improvement over the study period in fresh cycle live birth rates in continental Europe. Indeed, by 2010, continental Europe for the first time caught up to U.S. rates. Because continental European data represent an amalgam of many different nations, cumulative European data are the most difficult to interpret. While SET practice rose in Northern Europe [24], its utilization in the whole of Europe still appears lower than in Australia/New Zealand, Japan and Canada (Fig. 2a).

During the entire study period live birth rates in frozen-thawed embryo cycles were highest in the U.S. (Fig. 3b). Starting in 2009, live birth rates in frozen-thawed embryo cycles also increased rapidly in the U.S. This increase in live birth rates was far more pronounced in frozen-thawed than fresh cycles. These observations, however, have to be interpreted with caution due to increased utilization of embryo banking in the U.S. in recent years, [25] which may have distorted ART outcome reporting [2, 26].

Live birth rates with frozen-thawed embryos increased all over the world. Indeed, in many regions live birth rates in frozen-thawed embryo cycles either approached or surpassed those achieved in fresh ART cycles. For example, in Australia/New Zealand improvements in embryo cryopreservation along with increased utilization of embryo banking led over the past five years to increased live birth rates in frozen-thawed embryo cycles (from 18.3 to 23.4%), now matching in that region reported live birth rates following fresh embryo transfer [11]. Interestingly, while fresh ART live birth rates clearly improved in continental Europe, they did so to a much smaller degree in frozen-thawed embryo cycles.

### Perinatal outcomes

In their annual ART reports, Australia/New Zealand and Canada emphasize perinatal outcomes, including mortality, gestational ages and birthweights. Reports from Australia/New Zealand emphasize live born singletons at term, with normal birthweight as the main outcome metric for ART [11]. Increasing use of SET in Australia/New Zealand has been credited with reducing ART multiple births from 8.2% in 2009 to 5.6% in 2013. In 2013 Australia/New Zealand reported a multiple delivery rate of 5.6% of which 5.5% were twins. Preterm delivery rate was 16.6% (10.5% for singletons, 67.3% for twins and 92.3% for higher order multiples). Low birthweight was reported in 12.7% of infants (6.7% for singletons following SET and 7.3% following DET, 56.3% for twins and 97.4% for higher order multiples). Perinatal mortality rate was 12.4 deaths per 1,000 births (Additional file 2).

In 2012 Canada reported that 27% of infants were born from multigestational pregnancies, of which 26.3% were twins and 1.1% were triplets [12]. For all ART procedures preterm delivery rates were 16.5% for singletons, 69.3% for twins and 100% for triplets. Low birthweight was reported for 21% of infants (7.9% for singleton, 54.5% for twins and 96.2% for triplets). The total perinatal mortality rate was 1.0% per infant.

In 2012 among fresh autologous ART cycles the U.S., 73% of live births were singletons, 26% were twins, and 1% were higher order multiples [17]. U.S. preterm delivery rates in 2012 fresh cycles were 11.1% for singletons, 17.4% for singletons from multiple pregnancies, 57.8% for twins and 95.3% for higher order multiples. Low birthweight was reported for 8.6% of singletons, 15.6% for singletons from multiple fetal pregnancies, 56.1% for twins and 92.3% for higher order multiples. Multiple-infant births were particularly high among women under age 35. In these young patients, 2012 U.S. data demonstrate that live births were the highest (57%) following DET, though the highest rates of singleton live births were observed following SET.

In 2013 Latin America reported a multiple birth rate of 21.8%, of which 20.7% were twins and 1.1% were higher order multiples. Preterm births were reported for 7.5% of singleton, 36.6% of twin, and 65.5% of triplet deliveries [16].

In 2011 Europe reported a multiple delivery rate of 19.2% in fresh ART cycles, of which 18.6% were twins and 0.6% triplets. While in frozen-thawed ART cycles the multiple delivery rate was 13.2% of which 12.8% were twins and 0.4% triplets. The preterm delivery rate for Europe was 12.0% for singletons, 53.9% for twins and 91.4% for triplets [13]. For the U.K. it was in all ART procedures 9.0% for singletons, 57.8% for twins and 97.6% for triplets. In 2011 fresh autologous ART cycles the U.K. reported a 20.6% multiple pregnancy rate [13, 14].

A detailed analysis of changes in Japanese perinatal outcomes, associated with the country’s rapidly changing ART practice patterns, was recently published [21]. In 2012, 96.0% of all ART pregnancies were singletons, 4.0% were twins and 0.05% were higher order multiples. Low birthweight was recorded for 16.8% of infants conceived with fresh embryos and 13.8% of those conceived with frozen-thawed embryos. Since implementation of an SET policy in 2008, rates of perinatal mortality decreased from 0.7 to 0.4% in fresh cycles but did not change in frozen-thawed cycles, which now account for most live birth in Japan. Preterm delivery rates also decreased between 2007 and 2012 from 13.5 to 9.4% in pregnancies conceived with fresh embryos and from 12.7 to 9.6% in those conceived with frozen-thawed embryos. Additionally, caesarian delivery rates decreased from 35.6% in 2007 to 32.8% in 2012 in fresh cycles but did not change in frozen cycles [21].

## Discussion

This review evaluated longitudinal data to gain insight into ART practices and outcomes, and demonstrated considerable disparities in live birth rates between regions in the world. While over the last 10 years most regions demonstrated improvement, some showed no change and others demonstrated declines in ART live birth rates.

These findings, of course, reflect major differences in patient demographics, societal norms, local laws, and economics, which impact ART practices and could not be adjusted for in this analysis. Direct comparisons of data from various regions are also subject to differences in rigor of data reporting, collection and verification outlined above. Interpretations of here presented data should, therefore, be made with caution, and in consideration of these issues. The study is also somewhat incomplete in that it does not include the Middle East, large parts of Asia, and all of Africa. One Middle Eastern country, Israel, on a per capita basis performs the highest number of ART cycles of any country in the world [9]. We were unable to draw on appropriately published ART data in any of these regions.

The U.S. maintained throughout the decade the highest fresh cycle live birth rates compared to other regions. Continental Europe, however, for the first time appears to have caught up to the US in its fresh cycle live birth rates by 2010. The longstanding difference between US and European outcomes has been subject of scrutiny by European and US investigators [27–29]. A number of reasons for the differences have been suggested, and investigators have in the past expressed strong opinions on this subject. Confluence of U.S. and Continental European fresh cycle live birth rates now suggest increasing congruity of ART practice between the two continents.

Newly integrated practices into ART to a large degree involve methods of embryo selection, including blastocyst-stage transfer, PGS, cryopreservation of all embryos and subsequent frozen-thawed transfer, and the utilization of SET. These new practices, however, may not benefit all patient groups equally and in some, particularly poor prognosis patients, may, negatively affect outcomes [30].

Live birth rates with frozen-thawed embryos improved rapidly in the U.S. (Fig. 3b). Reported U.S. live birth rates may be somewhat inflated due to exclusion from national outcome reporting of embryo banking cycles, some PGS cycles, and cycles where no embryos are created for transfer or survive thaw to be transferred [2, 26].

In some regions, live birth rates with frozen-thawed embryos exceed those with fresh embryos. This might be interpreted as evidence in support of routine embryo banking in lieu of fresh transfer, [4, 31] especially since efficiency of cryopreservation has improved with vitrification. However, it more likely reflects differences in patient populations undergoing fresh versus frozen-thawed embryo transfer. For example, younger and favorable prognosis patients maybe more likely to have surplus embryos for cryopreservation or banking and therefore may be relatively overrepresented in frozen-thawed versus fresh cycles. Similarly, we have recently demonstrated how patient selection biases in US national data led CDC investigators to incorrect conclusions about PGS effectiveness [32].

In 2004 Japan already demonstrated the lowest fresh cycle live birth rates among all regions. Over the following decade, Japan almost tripled its number of ART cycles (Fig. 1a) and lost almost two-thirds of its fresh cycle live births, dropping to a national rate of 5% by 2012 (Fig. 3a). The loss in fresh cycle live births was partially compensated for by a relatively higher live birth rate in frozen-thawed embryo transfer cycles, resulting in a relatively stable annual total live birth rate (including fresh and frozen-thawed cycles) as recently reported by Takeshima et al [21]. Indeed, most ART infants in Japan in recent years were conceived in frozen-thawed rather than fresh ART cycles. Our findings along with those reported by Takeshima et al. suggest that a large increase in ART cycle volume was required to achieve a very modest improvement in perinatal outcomes and offset declining fresh cycle live birth rates.

No other region in the world demonstrated such rapid change in ART practice. As such, it appears that changes in national practice patterns in Japan, not equally experienced in other regions in the world, led to the observed outcome changes. For example, Teramoto and Kato proposed minimal stimulation IVF and by 2007 reported having performed 43,433 such cycles [7]. Similar protocols have since been adopted by many Japanese ART centers, and sporadically elsewhere [33–35]. The dramatic changes in Japanese ART cycle numbers and outcomes, likely represent a combination of minimal stimulation protocols, the potentially lower implantation rate in fresh versus frozen-thawed transfers due to endometrial factors, the fresh transfer of poor quality embryos that may not go on to blastocyst-stage, progressive migration to thawed embryo transfer, as well as, implementation of stringent SET regulations [21].

Embryo selection efforts have also gained followers elsewhere: For example, SET at blastocyst- stage has also become a characteristic feature of ART in Australia/New Zealand, [11, 36] areas of Canada [22] and in Northern Europe [37, 38]. Australia/New Zealand reported already in 2004 relatively low live birth rates in fresh ART cycles which gradually further declined as utilization of SET at blastocyst-stage increased (Fig. 3a). Australia and New Zealand progressively shifted from cleavage- to blastocyst- stage embryo transfers, with blastocyst-stage transfers increasing from 49.8% in 2009 to 61.1% in 2013. Concomitantly, fresh cycles that reached embryo transfer in Australia and New Zealand decreased from 76.6 to 67.5% [11]. Likewise, data from Canada demonstrate a decline in fresh cycle live birth rates following implementation of an SET mandate in the province of Quebec in 2009 [23]. Increased utilization of SET, therefore, appears to be temporally associated with declines in fresh cycle live birth rates. Regions with strict SET policies have been able to lower their multiple delivery rates, for example by 2012–2013 Australia/New Zealand and Japan reported the lowest multiple delivery rates of 5.6% and 4% while the US had the highest of 27%.

Utilization of elective SET is based on the premise that twin pregnancies increase maternal and neonatal outcome risks in comparison to singletons [39]. Our group considers that a more appropriate way of framing the question in infertility patients is to compare the outcome risks of two consecutive singleton pregnancies to one twin pregnancy [40, 41]. Moreover, it is important to recognize that the risk profiles of singleton and twin neonates vary greatly depending on whether conception occurred spontaneously or via ART [42, 43]. Nevertheless, to minimize risks of a twin pregnancies, elective SET followed by a frozen-thawed embryo transfer has in recent years been increasingly offered to good prognosis patients, as this approach is reported to produce a similar cumulative pregnancy chance to DET [44]. It is also important to note that we did not differentiate between elective and non-elective SET in this review because this classification is typically not discernable in registry reports. Patients undergoing non-elective SET are typically those with poor prognosis or patients undergoing minimal stimulation IVF, both groups likely experience somewhat lower live birth rates than good prognosis patients who are the best candidates for elective SET [34, 44–46].

Pregnancy and live birth rates have been the traditional metrics of ART success. More recently, investigators proposed that measures of neonatal health should also be incorporated [47, 48]. As revealed in this study, there are inconsistencies in the way perinatal outcome data are presented by various registries, making comparison between regions difficult. Further research appears indicated to develop better perinatal outcome associations with different ART practice patterns. Moreover, while most current ART registries present data per cycle of treatment, efforts are now underway in Australia/New Zealand and in the U.S. to link successive treatment cycles undertaken by each female patient. Therefore, ART registry reports may be evolving to define ART success as the birth of term, normal birthweight neonates per number of treated patients during the calendar year.

## Conclusion

In conclusion, our worldwide data analysis demonstrates increasing utilization of ART, SET, and of frozen-thawed embryos. Substantial differences in live birth rates exist among various regions of the world. Since ART practices affect different patient populations in varying ways, further research is needed to better define individual practices in different patient populations. Concomitantly, the profession should strive to achieve worldwide consensus as to what metric(s) define(s) outcome success in association with ART.

## Additional files

Additional file 1: Figure S1. PRISMA Flow Diagram. (DOC 30 kb)
Additional file 2: Table S1. Summary of prenatal outcomes for the most recent year of data. (DOCX 12 kb)

## Abbreviations

ART: Assisted reproductive technology
SET: Single embryo transfer
DET: Double embryo transfer
PGS: Preimplantation genetic screening
ICMART: International committee for monitoring assisted reproductive technology
ANZARD: Australia and New Zealand assisted reproduction database
NPESU: National perinatal epidemiology and statistics unit
CARTR: Canadian assisted reproductive technologies register
HFEA: Human fertilization and embryology authority
JSOG: Japan society of obstetrics and gynecology
RLA: Latin American registry of assisted reproduction
CDC: Centers for disease control and prevention
