# Supplementary material for: Systematic review of worldwide trends in assisted reproductive technology 2004–2013
Source: Reprod Biol Endocrinol. 2017 Jan 10;15:6. doi: 10.1186/s12958-016-0225-2 (PMC5223447; doi:10.1186/s12958-016-0225-2)
Supplement: Additional file 1: Figure S1. — PRISMA Flow Diagram. (DOC 30 kb) [file 12958_2016_225_MOESM1_ESM.doc]

**Additional file 1: Figure S1.**

**Screening**

**Included**

**Eligibility**

**Identification**

Records identified through database searching
(n = 34 )

Additional records identified through other sources
(n = 33)

Records after duplicates removed
(n = 67)

Records screened
(n = 67)

Records excluded
(n = 4)

Full-text articles assessed for eligibility
(n = 63)

Full-text articles excluded, with reasons
(n = 4)

Studies included in qualitative synthesis
(n = 59)

Studies included in quantitative synthesis
(n = 59)
