# Supplementary material for: Systematic review of worldwide trends in assisted reproductive technology 2004–2013
Source: Reprod Biol Endocrinol. 2017 Jan 10;15:6. doi: 10.1186/s12958-016-0225-2 (PMC5223447; doi:10.1186/s12958-016-0225-2)
Supplement: Additional file 2: Table S1. — Summary of prenatal outcomes for the most recent year of data. (DOCX 12 kb) [file 12958_2016_225_MOESM2_ESM.docx]

| **Region (year)** | **Preterm Delivery**  **(<37 weeks)** | **Low Birth Weight**  **(<2500 grams)** |
| --- | --- | --- |
| (% of deliveries) |  |  |
|  |  |  |
| **Australia and New Zealand (2013)** | **16.6%** | **12.7%** |
| singletons (94.4%) | 10.5% | 6.7% |
| twins (5.5%) | 67.3% | 56.3% |
| higher order (0.1%) | 92.3% | 97.4% |
| **Canada (2012)** |  | **21.0%** |
| singletons (84.3%) | 16.5% | 7.9% |
| twins (15.3%) | 69.3% | 54.5% |
| triplets (0.4%) | 100.0% | 96.2% |
| **Europe (2011)** |  |  |
| singletons (80.8%) | 12.0% |  |
| twins (18.6%) | 53.9% |  |
| higher order (0.6%) | 91.4% |  |
| **Japan (2012)** | **9.5%** | **14.6%** |
| singletons (96.0%) |  |  |
| twins (4.0%) |  |  |
| higher order (0.05%) |  |  |
| **Latin America (2012)** |  |  |
| singletons (78.2%) | 14.0% |  |
| twins (20.6%) |  |  |
| higher order (1.2%) |  |  |
| **UK (2011)** |  |  |
| singletons (79.4%) | 9.0% |  |
| twins | 57.8% |  |
| higher order | 97.6% |  |
| **US (2012)** |  |  |
| singletons (73%) | 11.1% | 8.6% |
| twins (26%) | 57.8% | 56.1% |
| higher order (1%) | 95.3% | 92.3% |
